# Controversies in Antimicrobial Stewardship: Focus on New Rapid Diagnostic Technologies and Antimicrobials

**DOI:** 10.3390/antibiotics5010006

**Published:** 2016-01-14

**Authors:** Eric Wenzler, Jordan R. Wong, Debra A. Goff, Christopher A. Jankowski, Karri A. Bauer

**Affiliations:** 1 College of Pharmacy, University of Illinois at Chicago, Chicago, IL 60612, USA; Wenzler@uic.edu; 2 Department of Pharmacy, Grady Health System, Atlanta, GA 30303, USA; JWong@gmh.edu; 3 Department of Pharmacy, The Ohio State University, Wexner Medical Center, Columbus, OH 43210, USA; Debbie.Goff@osumc.edu; 4 Department of Pharmacy, University of Florida Health Jacksonville, Jacksonville, FL 32209, USA; Christopher.Jankowski@jax.ufl.edu

**Keywords:** stewardship, antimicrobial, rapid diagnostic technology, outcomes

## Abstract

Antimicrobial stewardship programs (ASPs) are challenged with ensuring appropriate antimicrobial use while minimizing expenditures. ASPs have consistently demonstrated improved patient outcomes and significant cost reductions but are continually required to justify the costs of their existence and interventions due to the silo mentality often adopted by hospital administrators. As new technologies and antimicrobials emerge, ASPs are in a constant tug-of-war between providing optimal clinical outcomes and ensuring cost containment. Additionally, robust data on cost-effectiveness of new rapid diagnostic technologies and antimicrobials with subsequent ASP interventions to provide justification are lacking. As the implementation of an ASP will soon be mandatory for acute care hospitals in the United States, ASPs must find ways to justify novel interventions to align themselves with healthcare administrators. This review provides a framework for the justification of implementing a rapid diagnostic test or adding a new antimicrobial to formulary with ASP intervention, reviews approaches to demonstrating cost-effectiveness, and proposes methods for which ASPs may reduce healthcare expenditures via alternative tactics.

## 1. Introduction

Antimicrobial stewardship programs (ASPs) are designed to optimize antimicrobial prescribing in order to improve patient care, stem the tide of antimicrobial resistance, and reduce healthcare expenditures [1,2]. ASPs have proven their ability to achieve these goals through a variety of interventions including antimicrobial use guidelines, prescriber education, antimicrobial restriction, prospective audit and feedback, and implementation of rapid diagnostic technologies [2,3,4,5]. These interventions have documented up to 36% reductions in antimicrobial consumption, annual cost reductions of up to $900,000, decreases in the rates of *Clostridium difficile* infection, and improvements in the rates of clinical cure and mortality for various infectious disease syndromes [6,7,8,9,10,11,12,13,14].

Despite these well known benefits, only approximately half of hospitals in the United States and worldwide have implemented a structured ASP [15,16,17]. The primary barrier to implementation of an ASP consistently cited throughout the literature is a lack of personnel and/or funding for the program [15,16,17,18,19]. In the largest survey to date, only 15% of ASPs reported dedicated funding for members of the program [16]. It is unclear why ASPs experience difficulty in receiving appropriate funding and are forced to justify their existence in terms of cost containment when ASP-related cost reductions are extensively documented throughout the literature [2]. A recent supplement devoted to antimicrobial stewardship in *Clinical Infectious Diseases* states that ASPs are cost-effective and often pay for themselves through savings in both antimicrobial expenditures and indirect costs. The authors state “The cost benefits of antimicrobial stewardship make it an easy sell for hospitals, especially in an era of increasing cost constraints” [20]. As the National Action Plan for Combating Antibiotic-Resistant Bacteria [21] requires that all acute care hospitals in the US implement an ASP by the year 2020, the role of antimicrobial stewardship in optimizing clinical and economic outcomes is paramount.

One of the primary goals of hospital administrators is economic efficiency [22]. A recent survey of 41 US institutions with an ASP reported that hospital and pharmacy administrators view antimicrobial costs as the most important ASP outcome. In contrast, the appropriateness of antimicrobial use was selected by physicians specializing in infectious diseases as the most important stewardship metric [23]. Recently, the vigorous pursuit of cost-containment in the US has resulted in conflict between healthcare efficiency and optimal patient outcomes. The introduction of medical advancements, including rapid diagnostic technologies and antimicrobials, allows opportunities for ASPs to provide timely and effective management of infectious diseases. Simultaneously, these advancements result in financial strain by creating conflict in the ability to meet the institution’s fiscal goals and advance medical practice. This often results in the “silo” mentality through the consideration of specific expenses or resources separate from overall healthcare expenditures and resources [24].

We propose two primary reasons for this disconnect between ASP practitioners and hospital and pharmacy administrators. First, ASPs have not done an adequate job in consistent documentation of cost savings. Frequently, economic outcomes are provided as “soft” dollars or cost avoidance. Secondly, administrators often view ASPs’ cost savings in silos. For example, a direct correlation can be made between a decrease in an antimicrobial expenditure as a result of a restrictive policy by the ASP. In contrast, cost savings associated with improved outcomes as a result of a rapid diagnostic technology combined with ASP intervention is difficult to directly attribute to the ASP.

Cost-containment is imperative in the US at a time in which healthcare expenditures exceed $3 trillion [25]. Importantly, pharmaceutical agents, including antimicrobials, are a leading healthcare expense [26]. Pharmaceutical expenditures are identifiable and represent a target for cost-containment efforts. There are few constraints on the price of medications as evidenced by the substantial prices of the direct-acting antivirals for the treatment of Hepatitis C [27] and the dramatic price increase of pyrimethamine [28]. Congressional discussions regarding these issues are currently underway [29], but, until these issues are resolved, ASPs are tasked with determining ways to convince hospital and pharmacy administrators that select medications may be necessary for some patients despite an increased drug expenditure.

ASPs must also consider that infectious diseases guidelines are developed by field experts based on evidence and opinion. These decisions are rarely based on cost-effectiveness analyses. For example, the Infectious Diseases Society of America (IDSA) [30] guideline on the management of methicillin-resistant *Staphylococcus aureus* (MRSA) bacteremia proposes daptomycin doses of 8–10 mg/kg in certain patient populations, even though the FDA approved dose is 6 mg/kg [31]. This recommendation is based on the complexity of the infection in specific patient populations and expert opinion. The recommendation is not based on robust cost-effectiveness analysis. Hospital administrators may negatively view this recommendation from a cost-containment perspective and disagree with the use of higher daptomycin doses. These situations place ASPs in difficult positions by forcing them to justify the cost of more expensive antimicrobials based on guideline recommendations and not on antimicrobial expenditures.

The direct antimicrobial cost should not be the only consideration for ASPs and hospital administrators. A primary goal of an ASP is to reduce the spread of resistance, which is associated with its own inherent cost [32]. The rapidly growing problem of antimicrobial resistance has been forced into the hands of policy makers who often view problems based on economic burden and the cost-effectiveness of interventions to change them. Unfortunately, health economists have been unable to demonstrate that the incurred costs associated with antimicrobial resistance are substantial enough to prioritize it as a health precedent [33]. From an economic perspective, antimicrobial resistance has been viewed as an externality in that its effects are unlikely to be felt by either the consumer (the patient) or the supplier of the treatment (the pharmaceutical company). This results in neither group having an incentive to reduce the use of antimicrobials despite the significant impact on the overall welfare of society. Importantly, the major effects of resistance will be experienced by future generations who will have had no impact on the present decisions [34]. The economic externality of antimicrobial resistance must be included in economic evaluations to better inform the decision-making process [35].

ASPs can assist by encouraging international collaboration [36,37]. Additionally, ASPs can assist in the education of patients as to the appropriate use of antimicrobials with assistance from programs such as the Center for Disease Control and Prevention’s Get Smart: Know When Antibiotics Work campaign.

The subsequent sections will provide a guide to ASPs on the cost justification of novel interventions, including rapid diagnostic technologies and antimicrobials, in an effort to optimize clinical and economic outcomes. Secondly, the manuscript will discuss alternative ways in which ASPs may effectively demonstrate cost saving in order to receive continual administrative support.

## 2. Justifying Novel Interventions

### 2.1. Rapid Diagnostic Technologies

The advent of rapid diagnostic technologies has revolutionized the way ASPs are able to make an impact on patient outcomes. Given the overall lack of novel antimicrobial agents in the pipeline, ASPs are forced to use current antimicrobial agents in innovative and effective ways. As time to appropriate antimicrobial therapy has been shown to be the single most important predictor of mortality in patients with infectious disease syndromes [38,39,40,41], identifying the causative pathogen and its resistance mechanisms sooner is extremely valuable for the optimization of patient care. Currently, there are numerous rapid diagnostic platforms. A recent review by Bauer *et al.* highlights the characteristics and considerations of a variety of rapid diagnostic technologies in addition to published studies evaluating the impact of ASPs utilizing these technologies [42]. The following is a specific example of how the implementation of the Nanosphere^®^ Verigene Gram Negative Blood Culture (BC-GN) system was justified at the Ohio State University Wexner Medical Center for use by our ASP.

The BC-GN assay is an *in vitro* diagnostic test for the rapid detection and identification of Gram-negative bacteria and resistance markers from positive blood cultures. When combined with ASP intervention, the BC-GN assay has been shown to reduce to the time to optimal therapy by greater than 10 h [43]. The BC-GN assay is able to detect *Acinetobacter* spp., *Citrobacter* spp., *Enterobacter* spp., and *Proteus* spp. at the genus level, and *Escherichia coli*, *Klebsiella pneumoniae*, *K. oxytoca*, *Pseudomonas aeruginosa*, and *Serratia marcescens* at the species level, along with the resistance markers CTX-M, IMP, KPC, NDM, VIM, and OXA [44].

The initial step in evaluating and justifying this technology was to collaborate with the clinical microbiologists in determining the utility and costs associated with the test. Secondly, the initial costs associated with either purchasing or leasing the instrument were determined. We also determined the necessary space for the instrument. The complexity of the technology was evaluated to ensure that the microbiology technicians were able to perform the test without extensive training. Because the technology is designed to be on-demand, we decided that the microbiology laboratory would perform the test 24/7; although, importantly, ASP personnel would not be available 24/7 to communicate the results and provide recommendations to clinicians. After the decision was made to implement the technology, the clinical microbiologists completed a validation of the instrument. The cost per sample and the number of blood cultures positive for a Gram-negative bacteria were determined from the previous year. This allowed for an annual expense calculation that could be used to justify the cost of instruments based on clinical and microbiological outcomes. Finally, an estimation of the number of blood cultures positive for a Gram-negative organism with resistant determinants was determined, as these cultures led to potential ASP interventions. For example, the majority of extended-spectrum β-lactamase enzymes are a result of the CTX-M gene. Our empiric β-lactam based on the susceptibilities at our institution is either piperacillin/tazobactam or cefepime. We assumed that we would be able to perform interventions on all blood cultures positive for a CTX-M gene and provide appropriate antimicrobial therapy recommendations which would potentially result in improved clinical and economic outcomes.

After completing the internal review, we evaluated literature demonstrating reduced cost savings with the combination of rapid organism identification and ASP intervention. There were no published studies evaluating cost savings using the BC-GN assay at the time; therefore, we extrapolated findings from similar studies. Specifically, cost savings reported by Bauer *et al.* [7] were considered, as the study was performed at our institution, as was the study by Sango *et al.* [45], which used the BC-GN assay. The number of patients and associated cost savings in these studies were compared to previous estimates on the number of patients upon whom interventions could be performed by our ASP. We determined that similar improved patient outcomes and cost savings could be anticipated; therefore, the decision was made to implement the BC-GN assay in combination with ASP intervention. The information was presented to our institution’s Antibiotic Subcommittee and subsequently the Pharmacy and Therapeutics Committee for approval. Our ASP was responsible for implementing the BC-GN assay and completing education for all medical and pharmacy personnel. Our ASP is responsible for evaluating all patients with a positive blood culture for a Gram-negative organism identified by the BC-GN assay and providing recommendations on optimal therapy to clinicians. A 4-month pre- and post-implementation evaluation was also completed evaluating clinical and economic outcomes.

### 2.2. New Antimicrobials

In evaluating the cost-effectiveness of an ASP, all relevant cost decreases or increases in addition to the health benefits achieved must be qualified and quantified in order to understand whether a particular intervention offers value. Unfortunately, most ASP studies to date do not include all aspects of costs such as staff resources and time, diagnostic modalities, and other healthcare resources. A recent meta-analysis described the current ASP literature regarding the accuracy and effectiveness of the reported cost savings data [46]. Only two of the 36 studies performed a full cost-effectiveness analysis. Scheetz *et al.* used an analytical model with a decision tree to determine the cost-effectiveness of ASP interventions on the reduction of morbidity and mortality associated with nosocomial bacteremia [47]. The authors determined that utilizing an ASP to inform the most appropriate antibiotics to treat nosocomial bloodstream infections reduced hospital stay and mortality, and led to savings of $2,367 per quality-adjusted life-year gained. Brown and Paladino also used a decision tree model to assess the cost-effectiveness of a rapid diagnostic technology and appropriate antimicrobial prescribing compared with conventional microbiological identification [48]. They found that the implementation of a RDT that informed the correct selection of antibiotics in patients with methicillin-susceptible *S. aureus* and MRSA bacteremia resulted in improved outcomes including reduced mortality rates. While improving outcomes, the results showed that rapid testing allowing for early antimicrobial optimization was also associated with reduced costs, with a cost-effectiveness ratio of $820 per life-year saved. These studies were well designed and used current recommendations for the appropriate design of a cost-effectiveness analysis [49].

Other examples of cost-effectiveness analyses exist in the literature. Although the studies do not specifically involve ASPs, they provide tools for stewardship practitioners to justify proposed antimicrobial formulary additions or interventions. Bhavnani *et al.* analyzed the cost-effectiveness of daptomycin compared to vancomycin and gentamicin for successful clinical response in the treatment of MRSA bacteremia [50]. Data for the model was partly obtained from an open-labeled randomized trial in which successful clinical response for daptomycin was 44.4% compared to 31.8% with vancomycin and gentamicin (treatment difference 12.6%, 95% confidence interval −7.38% to 32.6%). The median cost-effectiveness ratio for drug acquisition cost was higher for daptomycin than vancomycin and gentamicin ($4,082 *vs.* $560, *p* < 0.001). Importantly, the median ratio for clinical success when considering drug costs, treatment failures, therapeutic drug monitoring, medication preparation, adverse drug events and hospital bed costs was not different between the groups ($23,639 *vs.* $25,668, *p* = 0.85). This study highlights the importance of considering global costs of patient care as opposed to only evaluating drug acquisition costs.

McComb and Collins determined the cost-effectiveness of empiric antimicrobial therapy for the treatment of enterococcal bacteremia, including vancomycin-resistant enterococci (VRE). The authors used values from published literature to develop a model that included the use of rapid diagnostic tests for early organism identification [51]. The cost of treatment was comparable for base case cost of life saved among ampicillin, vancomycin, daptomycin and linezolid ranging from $35,967–$36,775. Use of a VRE-active agent, including daptomycin or linezolid, was associated with an increased rate of survival by 7%. As a result, the incremental cost-effectiveness ratio was $11,703 and $11,084 for daptomycin and linezolid, respectively. Importantly, the authors determined that empiric treatment with ampicillin was less effective and therefore more expensive despite its low drug cost. This study exemplifies the need to consider the clinical and economic costs associated with inappropriate antibiotic therapy, and the substantial costs of negative outcomes, including preventable death.

Collins *et al.* evaluated posaconazole compared to fluconazole or itraconazole as prophylaxis for invasive fungal disease in neutropenic patients [52]. Inputs for their model were partly derived from published clinical trial data. The base case daily posaconazole cost was $82 *vs.* $37 for fluconazole and $42 for itraconazole. The cost-effectiveness ratio was $3,083 for posaconazole *vs.* $6,010 for fluconazole or itraconazole. The authors concluded that fluconazole or intraconazole-treated patients received less cost-effective therapy based on the improved clinical efficacy of posconazole.

For the majority of new antimicrobials, a cost-effectiveness analysis will not be available when ASPs are considering the agent for formulary. ASPs must evaluate the specific disease state for which the antimicrobial will be used. Our ASP completed a clinical and economic evaluation of fidaxomicin prior to its addition to formulary. We collaborated with clinical microbiologists and hospital epidemiologists to determine the number of patients positive for *Clostridium difficilie* via polymerase chain reaction (PCR). We further determined the number of patients with recurrent *Clostridium difficile* infection (CDI). The clinical trials on fidaxomicin determined that the agent was associated with decreased recurrences for patients infected with specific strains [53,54]. We selected institution specific criteria for the use of fidaxomicin as part of our CDI treatment guideline. We determined that, if we administered fidaxomicin to all patients with CDI, we would prevent a significant percentage of recurrences in which the institution would realize a significant cost savings. Our ASP presented the information to Antibiotic Subcommittee and subsequently the Pharmacy and Therapeutics Committee with the recommendation for a fidaxomicin formulary addition with associated criteria for use and ASP approval. Finally, our ASP collected data and analyzed clinical and economic outcomes in patients with CDI who received fidaxomixin [55].

Even with current literature evaluating cost-effectiveness, there are still opportunities for improvement. Published studies used data from previous studies often performed at other institutions to construct the model. Currently, the lack of consistency in reported cost outcomes in ASP-specific studies inhibits the ability to make robust comparisons between programs, resulting in a difficulty in selecting the most efficient, cost-effective strategy. In order to demonstrate the cost-effectiveness of an ASP to hospital and pharmacy administration, future studies must collect data on the effectiveness of their own interventions and incorporate the information into a decision tree model. This individualized data can provide powerful information to hospital administration prior to the implementation of a new intervention. Additional well-designed studies are needed along with a guideline developed by stewardship experts on how to justify their costs and produce meaningful data that can consistently promote and sustain ASPs.

### 2.3. Alternative ASP Strategies

If ASPs are to be successful, practitioners must change the perception that cost is the most important metric. The silo approach often taken by hospital administrators will only continue to antagonize a stewardship program’s efforts if the sole purpose of the program is to decrease the antimicrobial budget. If decreasing the antimicrobial budget remains the sole purpose, one must answer the question, how far can an ASP decrease the budget before patient care is compromised? To address this, stewardship programs must routinely meet with hospital and pharmacy administrators and obtain buy-ins from key stakeholders, both ID-trained and non-ID clinicians, regarding a rapid diagnostic technology or new antimicrobial. Further robust literature is needed to more accurately qualify and quantify the cost-effectiveness of ASPs. Furthermore, ASPs must look for new, alternative ways to demonstrate cost-effectiveness. In particular, ASPs must align themselves with initiatives that are essential to the operation and success of the institution.

It is imperative for ASPs to begin or continue to target interventions that are directly associated with reimbursement, financial penalties, and/or publicly reported outcomes [56,57]. As the healthcare environment shifts towards models that use payment incentives or financial penalties for exceeding or failing to achieve certain quality thresholds, ASPs are in a key position to focus efforts on these initiatives. Certain measures, including the Surgical Care Improvement Program (SCIP) are directly linked to value-based purchasing. ASPs are often tasked with creating, updating, and enforcing institutional guidelines for perioperative antimicrobial prophylaxis. ASPs have the opportunity to directly correlate interventions with costs and overall institutional performance. ASPs must collaborate with hospital administrators to identify important incentives and complete interventions that will positively impact these areas. By focusing efforts in these areas and demonstrating positive outcomes, ASPs may have the ability to perform less easily justifiable interventions on other infectious diseases states.

Healthcare administrators are often interested in benchmarking outcomes with other hospitals. ASPs can assist by benchmarking institutional antimicrobial use through the Center for Disease Control and Prevention’s Antimicrobial Use and Resistance and the National Healthcare Safety Network. These programs may allow the shift from solely antimicrobial cost to utilization. ASPs must also target The Joint Commission accreditation standards and communicate these standards to hospital administration. The Centers for Medicare and Medicaid also have several national quality indicators, including central-line associated bloodstream infections and pneumonia-associated mortality. By focusing on patient-safety activities, ASPs can demonstrate significant value through the avoidance of patient harm, which is often a tangible initiative. Finally, similar programs such as anticoagulation stewardship programs have been cited as reasons to defer ASP funding. ASP practitioners must continue to demonstrate and communicate their ability to significantly improve clinical and economic outcomes.

## 3. Conclusions

With the mandated implementation of ASPs in the US, the ability to optimize patient outcomes while simultaneously reducing healthcare expenditures will be imperative for stewardship practitioners. Through this paper, we have provided an overview of the problem and provided a framework for ASPs to address the challenges associated with rapid diagnostic technologies and new antimicrobials. We encourage additional publications by ASPs that demonstrate improved clinical outcomes while providing cost-effective care.
